# Anti-BCMA CAR-T cells for treatment of plasma cell dyscrasia: case report on POEMS syndrome and multiple myeloma

**DOI:** 10.1186/s13045-018-0672-7

**Published:** 2018-10-22

**Authors:** Jinhuan Xu, Qiuxiang Wang, Hao Xu, Chaojiang Gu, Lijun Jiang, Jue Wang, Di Wang, Bin Xu, Xia Mao, Jin Wang, Zhiqiong Wang, Yi Xiao, Yicheng Zhang, Chunrui Li, Jianfeng Zhou

**Affiliations:** 1 Department of Hematology, Tongji Hospital of Tongji Medical College, Huazhong University of Science and Technology, 1095 Jie-Fang Avenue, Wuhan, 430030 Hubei People’s Republic of China; 2 Immunotherapy Research Center for Hematologic Diseases of Hubei Province, 1095 Jie-Fang Avenue, Wuhan, 430030 Hubei People’s Republic of China; 3 College of Life Science and Health, Wuhan University of Science and Technology, Wuhan, 430065 Hubei People’s Republic of China

**Keywords:** Chimeric antigen receptor T cells, B cell maturation antigen, Remissions, Multiple myeloma, POEMS syndrome

## Abstract

**Background:**

POEMS (polyneuropathy, organomegaly, endocrinopathy, monoclonal gammopathy, and skin changes) syndrome still has no standard treatment. On the basis that both POEMS syndrome and myeloma have an underlying plasma cell dyscrasia, anti-myeloma therapy can be expected to be useful for POEMS syndrome. Chimeric antigen receptor T (CAR-T) cells targeting B cell maturation antigen (BCMA) has been used in the treatment of relapsed and refractory multiple myeloma (RRMM). No POEMS syndrome cases treated with anti-BCMA CAR-T cells have been reported.

**Case presentation:**

Here, we, for the first time, report a POEMS syndrome case treated with anti-BCMA CAR-T cell**s**. A 49-year-old female with incapacitating POEMS syndrome that progressed on lenalidomide treatment was enrolled in a phase I study involving anti-BCMA CAR-T cell**s** (ChiCTR-OPC-16009113). Another patient with RRMM who had undergone six prior lines treatments was also enrolled in the study. They received infusions of anti-BCMA CAR-T cells. Both patients achieved a stringent complete response. Complete remission persisted in the patient with POEMS syndrome and lasted for 7.6 months before a relapse in RRMM patient. Both patients had toxicity consistent with the grade 1 cytokine release syndrome.

**Conclusions:**

This is the first report of treatment by anti-BCMA CAR-T cells in POEMS syndrome. Our findings demonstrate the anti-BCMA CAR-T cell treatment may be a feasible therapeutic option for patients with POEMS syndrome and RRMM who do not respond well to traditional therapies.

**Trial registration:**

ChiCTR-OPC, ChiCTR-OPC-16009113. Registered 29 August 2016.

**Electronic supplementary material:**

The online version of this article (10.1186/s13045-018-0672-7) contains supplementary material, which is available to authorized users.

## Introduction

New treatment strategies for multiple myeloma (MM), a malignant hematopathy derived from plasma cells, are yet to be developed due to increasing prevalence of refractory/relapsed cases showing resistance to conventional therapy [1–4]. The POEMS syndrome, characterized by a collection of polyneuropathy, organomegaly, endocrinopathy, myeloma protein, and skin changes, is a rare paraneoplastic disorder caused by plasma cell dyscrasia, with poor prognosis and lack of standard treatment. Recent studies have shown that therapy by chimeric antigen receptor T (CAR-T) cells had a good effect on malignant hematological diseases including MM [5–10]. Carpenter et al. for the first time reported in vitro and in vivo anti-myeloma effects of anti-BCMA CAR-T cells [11]. Succeeding clinical investigations taking anti-BCMA CAR-T cells against relapsed/refractory MM (RRMM) have observed improved response and possibly curable effects [9, 10]. Considering the close similarity in pathology and potential efficacy of anti-myeloma regimens against POEMS can be expected a treatment effect by anti-BCMA CAR-T cells in subjects with POEMS [12, 13]. In this report, two cases with plasma cell dyscrasia (a POEMS and an RRMM) treated with anti-BCMA CAR-T cells are presented and reviewed the literature on clinical trials concerning anti-BCMA CAR-T cells in MM.

## Case presentation

### Case 1

**Fig. 1:**
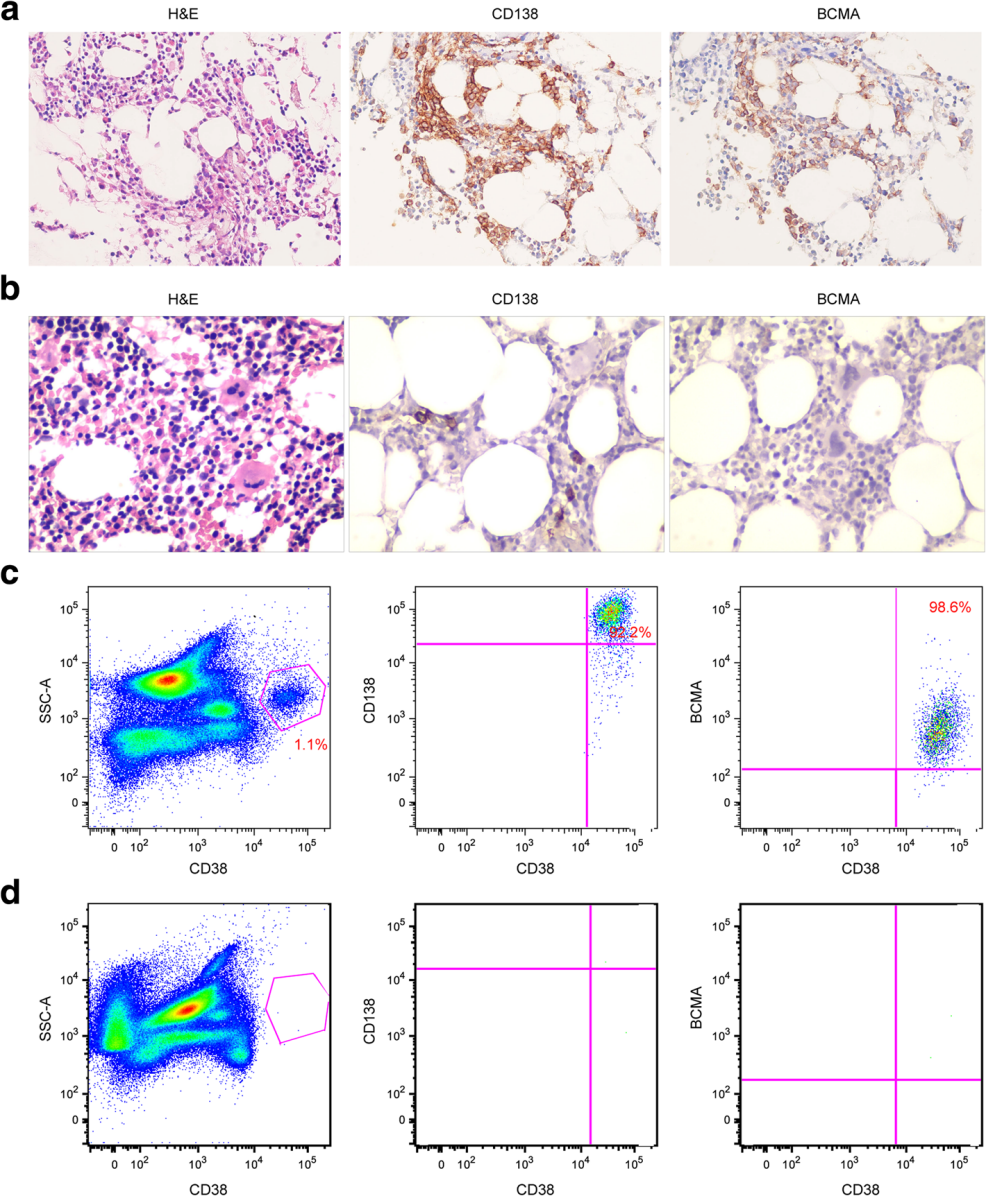
Bone marrow core biopsy and cell samples obtained before and after anti-BCMA CAR-T cells infusion **a** Hematoxylin and eosin staining, Immunohistochemical (IHC) staining for CD138 and BCMA. Bone marrow cells were 3% plasma cells as shown by CD138 staining at 7 days before the anti-BCMA CAR-T cells infusion. BCMA expression was uniform on the CD138 positive plasma cells (original magnification, × 400). **b** No plasma cells on hematoxylin and eosin staining, CD138, and BCMA immunostaining on day 60 after anti-BCMA CAR-T cells infusion (original magnification, × 400). **c** Flow cytometry showed uniform BCMA expression on CD38-positive malignant plasma cells before the anti-BCMA CAR-T cells infusion. **d** No plasma cells in the bone marrow cells on day 60 after anti-BCMA CAR-T cells infusion

A 49-year-old female presented to our hospital in September 2016 due to numbness and weakness in both lower extremities for 2 weeks. Because of progressive weakness involving both lower limbs, the patient became disabled with unstable gait, unable to walk independently. Physical examination showed 4-grade muscle strength of both lower legs. After admission, detailed examinations were performed. Electromyogram revealed findings consistent with an acquired demyelinating polyneuropathy with increased distal latencies, conduction blocks, prolonged F wave latencies, and reduced conduction velocity. The cerebrospinal fluid examination was normal. Ultrasonography detected splenomegaly, ascites, pericardial effusion, and pleural effusion. No signs of sclerotic or lytic lesions were noted in X-ray or CT scans. Blood cell count and clinical biochemistry examinations were normal. Serum monoclonal protein was detected by serum protein electrophoresis. A largely elevated serum vascular endothelial growth factor (VEGF) level (2350.0 pg/mL) was detected by enzyme-linked immunosorbent assay (ELISA). Decreased cortisol and thyroxine level was recorded. Bone marrow aspiration and biopsy identified monoclonal plasma cells. According to immunohistochemistry (IHC) analysis, plasma cells (CD138^+^) accounted for 3%, and BCMA and CD138 expression was highly concordant (Fig. 1a). 1.1% of cells were considered monoclonal plasma cells in flow cytometry (Fig. 1c). According to the latest diagnostic criteria for POEMS [14, 15], the diagnosis of POEMS syndrome, in this case, was established based on the following evidence: major items including polyneuropathy, monoclonal plasma cell neoplasm, and elevated serum VEGF and minor items including splenomegaly, endocrine abnormality, and extravascular volume overload.

**Fig. 2:**
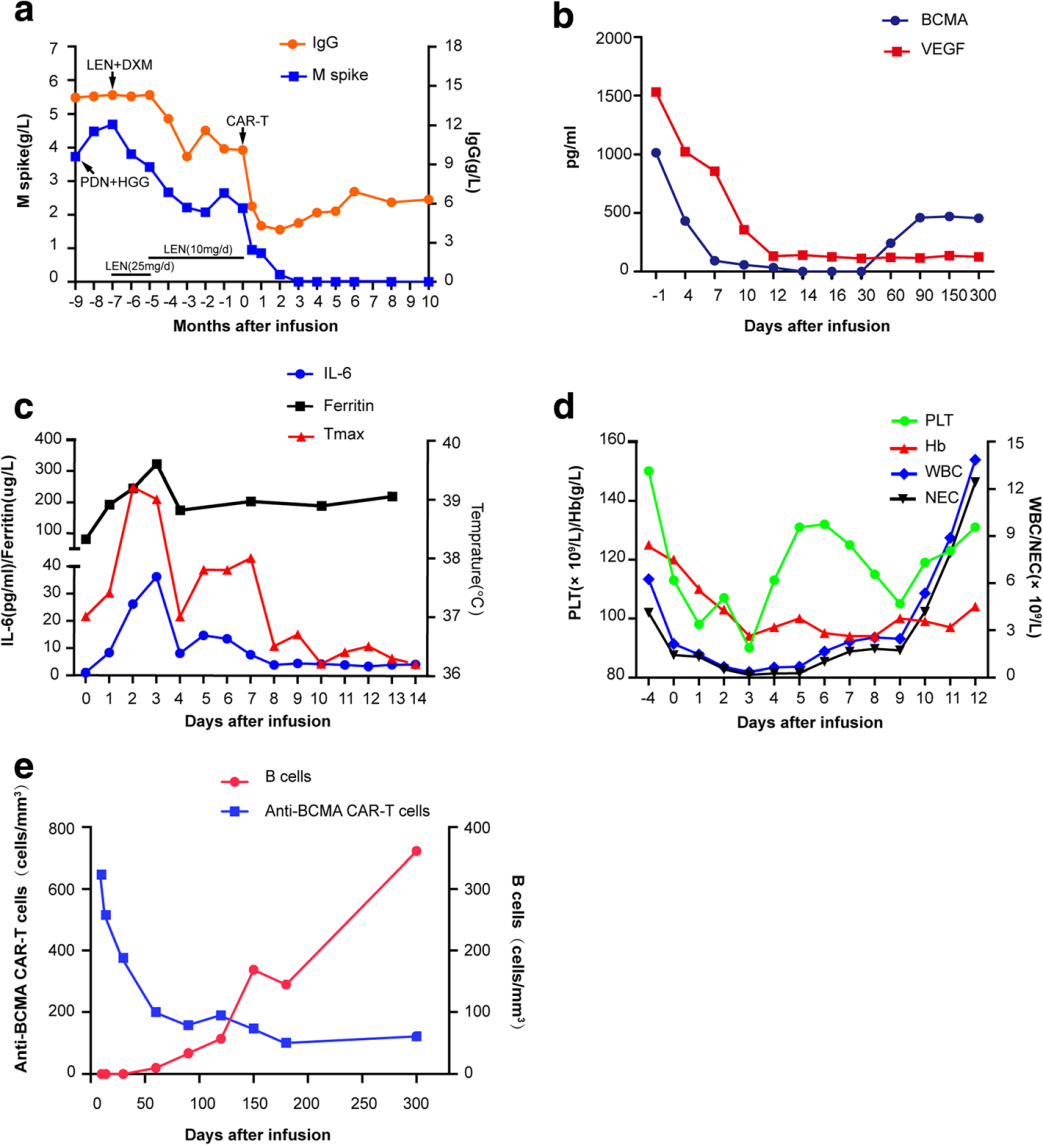
Measures of POEMS syndrome burden and clinical responses to infusions of anti-BCMA CAR-T cells. **a** The trend in IgG and M spike concentrations on the course of the all treatment, with PDN combined HGG, with LEN combined DXM and anti-BCMA CAR-T cells infusion. **b** Serum soluble BCMA and VEGF of the patients was measured by ELISA before, and after anti-BCMA CAR-T cells infusion, they both decreased post-treatment obviously. **c** Twenty-four hours after anti-BCMA CAR-T cells infusion, the patient became febrile. She was febrile for 7 days. The plot shows the maximum temperature for each day. Serum levels of cytokines were measured at the indicated time points. Levels of IL-6 and ferritin elevated markedly on day 3 and then dropped quickly. **d** Changes in the white blood cell and platelet count, hemoglobin level. **e** Anti-BCMA CAR-T cells engraftment, measured by means of flow cytometry as the number of cells per cubic millimeter, and the corresponding B cell frequencies, measured as the number of cells per cubic millimeter (in peripheral blood)

**Fig. 3:**
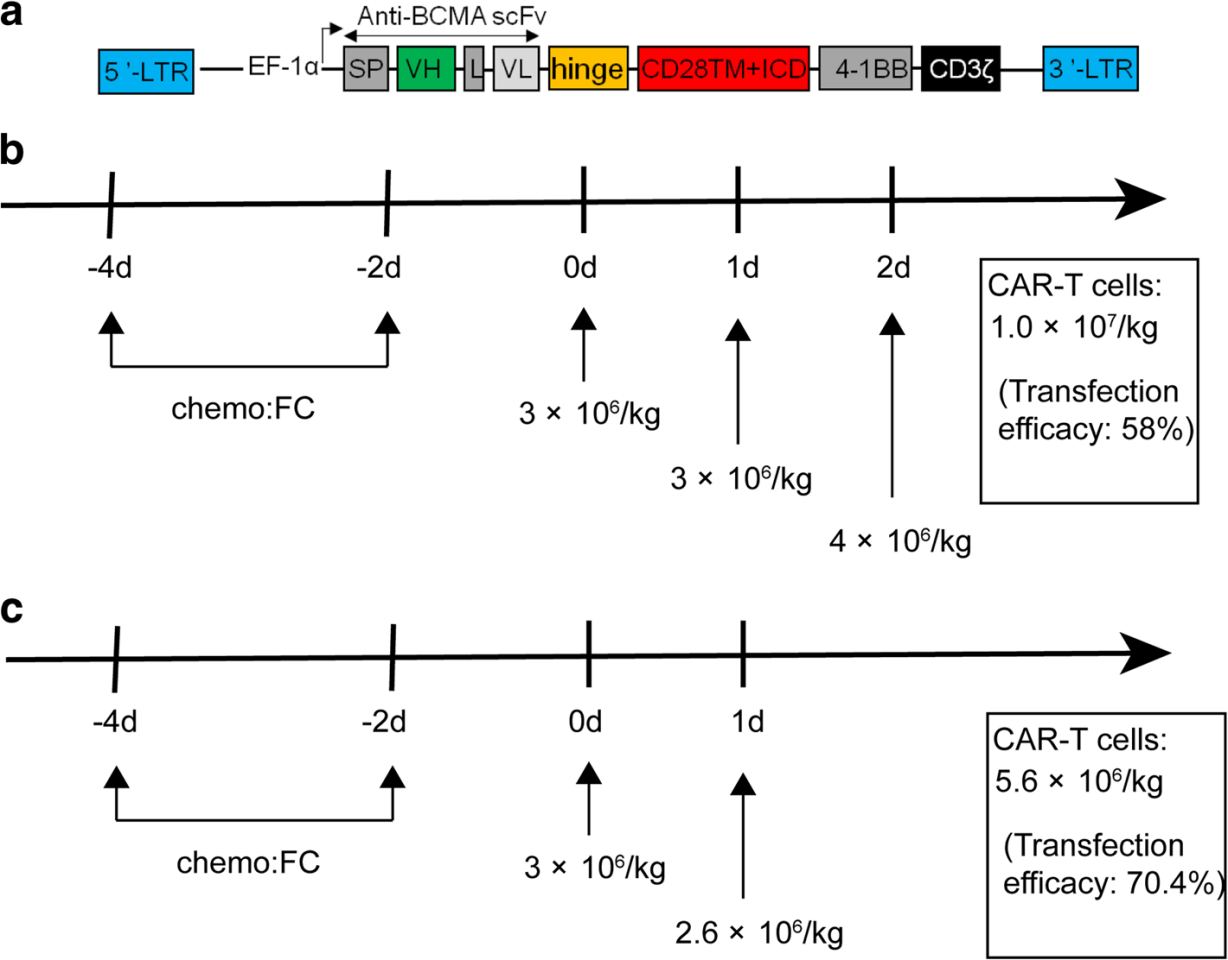
Construction of BCMA-specific CAR and protocol of anti-BCMA CAR-T cell infusions following chemotherapy. **a** Schematic diagram of anti-BCMA CAR vector. SP signal peptide, VH variable H chain, L linker, VL variable L chain. A protocol of CAR-T infusion in combination with chemotherapy. Chemotherapy included fludarabine and cyclophosphamide. CAR-T cells were infused at a total dose of 1 × 10^7^/kg for 3 days (**b** a patient of POEMS syndrome) and 5.6 × 10^6^/kg for 2 days (**c** a patient of multiple myeloma)

We started treatment with lenalidomide (25 mg/day for 21 days of a 28-day cycle) and once-weekly dexamethasone (10 mg). Serum monoclonal protein drastically decreased and neuropathy relieved after treatment for two cycles; however, frequent episodes of treatment-related orthostatic hypotension resulted in dose reduction of lenalidomide (10 mg/day for 21 days of a 28-day cycle) for the next 3 months. The serum monoclonal protein was largely reduced, and walking difficulty alleviated. However, the serum monoclonal protein quickly re-augmented, and symptoms worsened as low-dose treatment continued (Fig. 2a). The patient refused recommended salvage treatment with the bortezomib-based regimen or autologous stem cell transplantation (ASCT). Considering the reported anti-myeloma effects of anti-BCMA CAR-T cells [7, 8] and abundant expression of BCMA on monoclonal plasma cells (98.6% as revealed by flow cytometry) (Fig. 1c) and elevated serum BCMA (Fig. 2b) in this patient, we encouraged her to participate in a clinical trial involving anti-BCMA CAR-T treatment, to which she agreed. Chemotherapy (fludarabine 25 mg/m^2^ and cyclophosphamide 20 mg/kg) was administered 4, 3, and 2 days before the first infusion of CAR-T cells (June 25, 2017) which was followed by two infusions administered once daily for the next 2 days. The effective anti-BCMA CAR-T cells totaled /1 × 10^7^/kg (Fig. 3b).

The patient developed a fever (37.4 °C, peaked 39.2 °C on the next day, and lasted for 7 days) and tachycardia about 24 h after the first infusion. The peak of serum ferritin and IL-6 (17.5 times higher than baseline) was detected on the third-day post-infusion, which was just the time where toxicity culminated (Fig. 2c). Cytopenia was observed but not central nervous system (CNS) toxicities. White blood cells and neutrophils returned to normal on the tenth day after infusion. No infusion of red blood cells or platelets was required during the entire treatment period (Fig. 2d). All coagulation and biochemistry parameters remained normal over the treatment period. She experienced grade 1 cytokine release syndrome (CRS), which was graded as described [16, 17]. The CAR-T cells remained detectable, and B cells gradually returned to normal levels in the blood 10 months after infusion (Fig. 2e).

Her serum monoclonal protein started to decrease 15 days after CAR-T cells infusion and became undetectable 90 days after infusion (Fig. 2a). The soluble BCMA and VEGF levels also decreased in a short time after treatment (Fig. 2b). Sixty days post-infusion, no abnormal plasma cells were found by bone marrow aspiration or flow cytometry (Fig. 1b, d), and she was able to do some cooking and walk without difficulty. An electromyogram showed that the conduction velocity of bilateral tibial nerves, peroneal nerves, sural nerves, and superficial peroneal nerves recovered, and the sensory nerve action potential (SNAP) amplitude was in normal range. F waves from bilateral tibial nerves showed a normal latency of 26.7 ms.

### Case 2

**Fig. 4:**
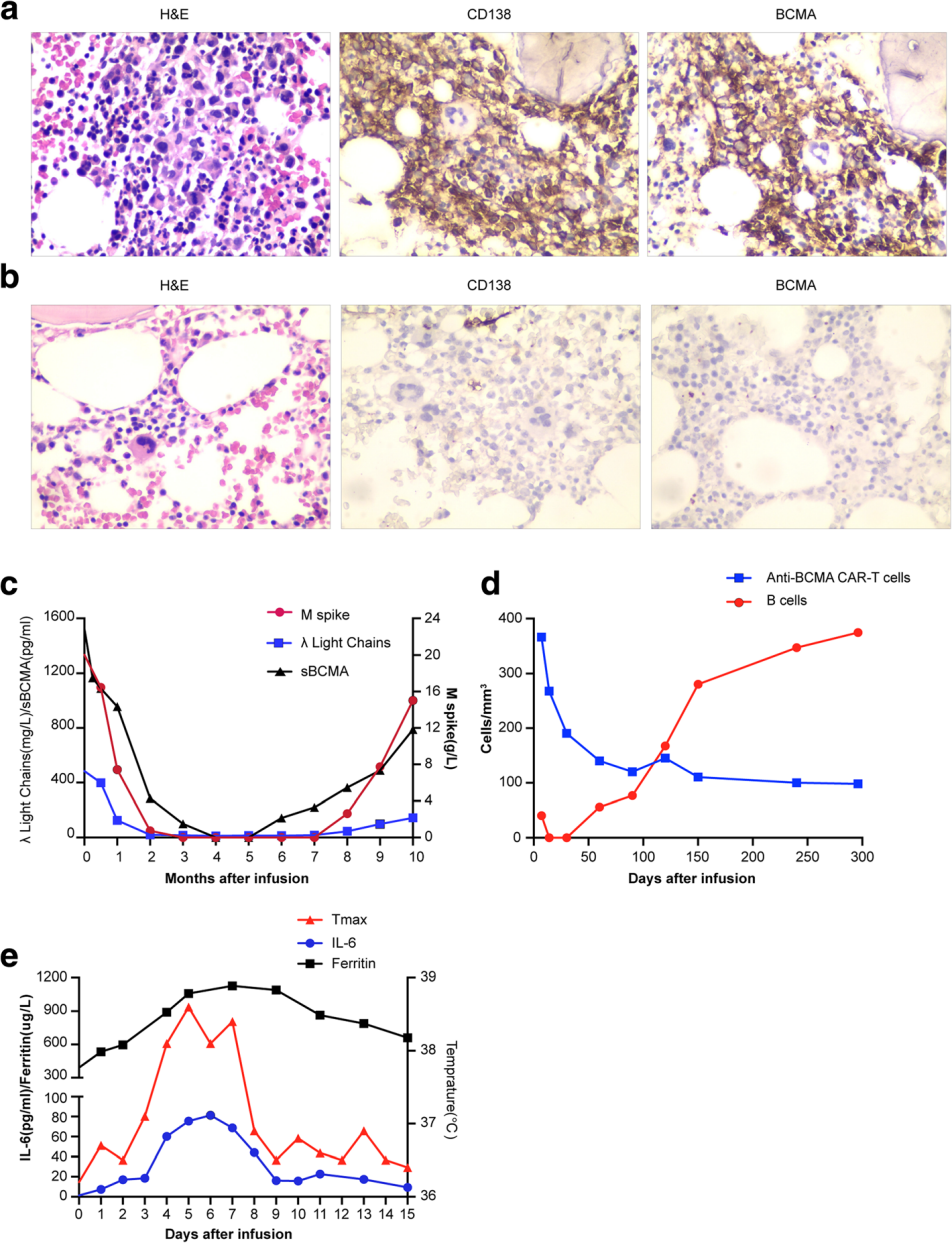
Measures of multiple myeloma burden and clinical responses to infusions of anti-BCMA CAR-T cells. **a** Before protocol treatment, the patient had a hypercellular bone marrow (hematoxylin and eosin). Bone marrow cells were 25% plasma cells as shown by CD138 staining. BCMA expression was obvious (original magnification, × 400). **b** Three months after CAR-T cell infusion, bone marrow plasma cells were completely absent as shown by the negative CD138 and BCMA staining (original magnification, × 400). **c** The trend in the patient’s serum M spike, free light chains, and BCMA concentrations after anti-BCMA CAR-T cells infusion, all of which decreased obviously. In the eighth month, these indicators began to rise. **d** Anti-BCMA CAR-T cells engraftment, measured by means of flow cytometry as the number of cells per cubic millimeter, and the corresponding B cell frequencies, measured as the number of cells per cubic millimeter (in peripheral blood). **e** 72 h after CAR-BCMA infusion, the patient became febrile. She was febrile for 7 days. The plot shows the maximum temperature for each day. The levels of IL-6 and ferritin was elevated

The patient received a diagnosis of IgA lambda multiple myeloma in November 2012, at the age of 59 years, after presenting with anemia and multiple bone lesions. She received induction therapy with 4 cycles of cyclophosphamide, bortezomib, and dexamethasone (CyBorD) which resulted in complete remission and high-dose melphalan (200 mg/m^2^ of body surface area) followed by ASCT as consolidation. Approximately 27 months later, the disease progressed, and 62% of her bone marrow cells were plasma cells. Subsequent treatment included regimens incorporating clarithromycin, lenalidomide, bortezomib, dexamethasone, and daratumumab. According to the International Myeloma Working Group (IMWG) response criteria [18], the response was poor, and the disease kept progressing. In August 2017, after the failure of six prior lines of treatment, the patient was enrolled in the aforementioned trial and received anti-BCMA CAR-T cells. A bone marrow biopsy showed a 25% myeloma involvement. CD138 and BCMA co-expression were revealed by IHC (Fig. 4a). Largely elevated serum soluble BCMA was detected by ELISA (Fig. 4c). Risk factors were noted, including a complex karyotype, t (4;14) translocation, and gain of 1q21. The treatment protocol was the same as case 1’s except that only two infusions of CAR-T cells were given and the effective cells totaled 5.6 × 10^6^/kg (Fig. 3c). The patient was febrile for 7 days with a maximum temperature of 38.6 °C (Fig. 4e). The serum ferritin and IL-6 peaked respectively on the seventh- and sixth-day post-infusion, which was also the time where toxicity culminated (Fig. 4e). Pancytopenia was observed, and there was no CNS toxicity. White blood cells and neutrophils returned to normal 12 days after infusion. The patient was platelet transfusion dependent from day 2 until day 21 after CAR-T cell infusion. All coagulation and biochemistry parameters were normal over the whole treatment period. She experienced grade 1 CRS. The CAR-T cells remained detectable, and B cells gradually returned to normal levels in the blood 10 months after infusion (Fig. 4d).

Serum monoclonal protein and free lambda (λ) light chain levels substantially decreased 15 days after treatment. Serum monoclonal protein became undetectable 90 days post-infusion and remained undetectable for the next 5 months (Fig. 4c). Soluble BCMA level in the serum also decreased sharply in a short time post-infusion (Fig. 4c). Bone marrow MM cells were not detected by bone marrow aspiration and CD138 staining 90 days after CAR-T cell infusion (Fig. 4b). The response to treatment was stringent CR. At the about 8th month, serum monoclonal IgA-λ recurred (Fig. 4c), which indicated MM relapse, but the patient was free of abnormal routine blood test findings and bone pain. Serum monoclonal protein and free λ light chain levels kept increasing as MM progressed and serum BCMA protein level as well (Fig. 4c). She had biochemical recurrence 7.6 months after treatment. However, the patient refused further bone marrow examination.

#### CAR-T cell production

Autologous peripheral blood mononuclear cells (PBMCs) were cultured with an anti-CD3 monoclonal antibody to induce T cell proliferation. The anti-BCMA CAR was encoded by a lentiviral vector containing a murine anti-BCMA single-chain variable fragment, a CD8a hinge, the CD28 transmembrane regions and intracellular domain, and CD3- ζ T cell activation domain (Fig. 3a). The anti-BCMA CAR-T cells were cultured for 14 days before infusion.

## Discussion

Our work demonstrated that anti-BCMA CAR-T cell treatment has a significant effect in patients with plasma cell dyscrasia, i.e., POEMS syndrome and RRMM. The POEMS syndrome case we describe had a rapidly progressing and disability-causing neurological disease that showed poor response to conventional therapy. She responded favorably to anti-BCMA CAR-T cells therapy. To the best of our knowledge, this is the first case report of anti-BCMA CAR-T cell treatment for POEMS syndrome. The patient had a dramatic improvement in neuropathy and functional status. Ten months after infusion, the anti-BCMA CAR-T cells were still detectable, and the number of B cells returned to normal levels in peripheral blood. An extended duration of CR without any treatment is expected, and the patient’s immune system recovered steadily. Another primary concern is the safety of anti-BCMA CAR-T cell treatment for POEMS syndrome. In this case, the patient developed grade 1 CRS. During the procedure, red blood cells and platelets were not infused, and white blood cells recovered quickly, which significantly reduced the risk of infection in the patient.

The study also suggested that anti-BCMA CAR-T cells have substantial anti-MM activity. A female patient with RRMM achieved CR after infusion of anti-BCMA CAR-T cells. The patient presented grade 1 CRS, which was characterized by fever and nausea. However, MM recurred with disease-free survival of 7.6 months. Our results were consistent with published studies [9, 10]. MM has been shown to be a phenotypically heterogeneous malignancy, with numerous sub-clones within the same patient [19–21], which may increase the risk of failure from single-antigen therapies; another cause of recurrence may be a loss of BCMA expression on malignant plasma cells. Because the patient refused further bone marrow examination, the proportion of malignant plasma cells and the expression of BCMA in the bone marrow were unclear. Ongoing efforts are attempting to make anti-BCMA CAR-T therapy more potent, safe, and affordable for patients. The CAR-T therapies are being developed to overcome relapse due to a reduced tumor antigen, including modification of T cells with two distinct CAR molecules with two different binding domains or one CAR molecule with two different binding domains in tandem [22–24].

It has been recently shown that serum BCMA levels correlated with clinical status and tumor burden of MM patients [9, 10, 25]. In our study, serum BCMA in both patients was largely elevated before anti-BCMA CAR-T cell infusion, decreased substantially after treatment, and maintained a lower level during disease remission. As the disease recurred and progressed, the serum monoclonal protein and free light chains started to increase, so as serum BCMA. Our results confirmed previous findings [25] and validated that serum BCMA level may be a reliable marker for monitoring and outcome prediction in MM patients [26].

Anti-BCMA CAR-T cell therapies have shown impressive anti-myeloma activities (some reaching 90–100%) in certain preclinical and/or clinical investigations (see Table 1), which is reviewed below.

**Table 1:** Clinical trials of anti-BCMA CAR-T cells for MM

Institution	NCI	Upenn	Bluebird Multi-Inst (Bb2121)	Nanjing Legend (LCAR-B38M)	MSK
Scfv derived from	Murine hybridoma	Human library	Murine hybridoma	Murine hybridoma	Human library
Co-stimulatory domain	CD28	4-1BB	4-1BB	4-1BB	4-1BB
Gene transfer	Retrovirus	Lentivirus	Lentivirus	Lentivirus	Retrovirus
Conditioning	Cy + Flu	Cohort 1: none Cohorts 2 and 3: Cy	Cy + Flu	Cy	Cy + Flu
BCMA Ag required	> 50%	No requirement	> 50%	“Clear expression”	> 1%
ClinicalTrials.gov identifier/reference	NCT02215967^9,10^	NCT02546167^27^	NCT02658929^28^	NCT03090659^29^	NCT03070327
Median prior lines	7	9	7	3	Not yet reported
Accrual	Completed (26 patients)	Completed (24 patients, data reported)	Ongoing (21 patients, data reported)	Ongoing (19 patients, data reported)	Not yet reported
Response	13 Of 16 (81%) ORR at highest dose	6 Of 10 (60%) ORR at high dose with Cy conditioning	17 Of 18 (94%) ORR at higher doses	19 Of 19 (100%) ORR	Not yet reported
Elimination gene	No	No	No	No	Truncated EGFR

*NCI* National Cancer Institute, *UPenn* University of Pennsylvania, *MSK* Memorial Sloan Kettering, *scFv* single chain variable fragment, *Cy + Flu* cyclophosphamide+ fludarabine

The National Cancer Institute (NCI) investigators for the first time published pre-clinical data on MM treatment using anti-BCMA CAR-T cells [9, 10]. Twenty six heavily treated MM patients were enrolled. They have continued to conduct a dose escalation trial demonstrating the potential for dramatic responses induced by this treatment modality for MM. Of the 10 patients treated with lower doses of anti-BCMA CAR-T cells (0.3–3.0 × 10^6^/kg), one patient experienced a very good partial response (VGPR) that lasted 8 weeks, eight patients had a stable disease that lasted from two to 12 weeks, and one patient had a transient partial response (PR). Of 16 patients treated at the highest dose level (9 × 10^6^ CAR-T cell/kg), 13 had PR or better response. The overall response rate for patients receiving 9 × 10^6^/kg was 81%, with 63% as VGPR or CR. Median event-free survival was 31 weeks. Treatment toxicity was mild at lower dose levels, with no CRS of grades 3 or 4 at cell doses of 0.3 to 3 × 10^6^/kg. At a cell dose of 9 × 10^6^/kg, CRS-related toxicities were substantial.

Cohen et al. [27] also reported their initial findings from a phase I trial at the University of Pennsylvania to explore the effects of anti-BCMA CAR-T cells in RRMM. This study used an anti-BCMA CAR with fully human antibody variable regions. The patients included in this trial were not screened for BCMA expression. Three cohorts were enrolled sequentially, and the objective was to collect preliminary data about safety, efficacy, and kinetics of expansion with the following protocols: (1) CAR-T cells alone at a dose of 1 to 5 × 10^8^/kg, (2) 1.5 g/m^2^ of cyclophosphamide with 1 to 5 × 10^7^/kg CAR-T cells, and (3) 1.5 g/m^2^ of cyclophosphamide with 1 to 5 × 10^8^/kg CAR-T cells. CAR-T cells were given as split-dose infusions. Twenty-four patients (median prior lines of therapy were seven) were evaluable; 96% had high-risk cytogenetics, including 71% with del(17p) or TP53 mutation, and a median 70% plasma cells on bone marrow biopsy. In cohort 1 (nine patients), four patients (44%) achieved ≥ PR response after CAR-T singlet treatment. In cohort 2, only one (20%) of five patients responded to the combination of cyclophosphamide and a tenfold lower dose of CAR-T, and this cohort was stopped early. In cohort 3, incorporation of cyclophosphamide with the higher dose (i.e., 1 to 5 × 10^8^) of CAR-T cells led to a disease response in six (60%) of ten patients. The median duration of response was 4 months, and there were ongoing responses in four patients (range, 3 to 24 months). Toxicities were similar with those reported in earlier studies, with severe CRS in 33% (8 of 24 patients) and severe neurotoxicity in 12.5% (3 of 24 patients).

Berdeja et al. [28] reported updated data about the dose escalation part of an anti-BCMA CAR-T multicenter trial conducted by bluebird bio. Four dose levels (50, 150, 450 and 800 × 10^6^ CAR-T cells) were explored in 21 patients with RRMM (median prior lines of therapy was 7). Responses were seen in 18 (86%) of 21patients, including 17 (94%) of 18 patients treated at doses of 150 × 10^6^ CAR-T cells or higher. Ten patients achieved PR (seven confirmed). With a median follow-up of 40 weeks, only four patients who experienced a response had subsequent progression, and the median progression-free survival was not reached; five patients had ongoing responses for more than 1 year. No treatment-related grade 3 or higher neurotoxicities were observed. CRS was primarily grade 1 or 2.

Nanjing Legend is conducting a trial in China with exciting early results [29]. A total of 19 patients with RRMM were enrolled in the trial. The median number of infused LCAR-B38M CAR-T cells was 4.7 (0.6 ~ 7.0) × 10^6^/kg. The median follow-up was 208 (62 ~ 321) days. A 100% objective response rate (ORR) was observed in RRMM patients. Eighteen out of 19 (95%) patients reached CR or VGPR status without myeloma-related biochemical and hematologic abnormalities in a median follow-up of 6 months. The majority (*n* = 14) of the patients experienced mild or manageable CRS, and the rest (*n* = 5) were even free of diagnosable CRS. This trial differs from the others in that the patients were treated at a significantly earlier phase in their treatment course, with median prior lines of therapy being 3, compared to 7–9 in other trials reported.

In addition to the trials described here, other trials of anti-BCMA CAR-T cells [30], including three in combination with other CAR-T cells, have been opened since March 2018. Initial data are expected by late 2018 or early 2019.

## Conclusions

The anti-BCMA CAR-T cell treatment may be a feasible therapeutic option for patients with POEMS syndrome and RRMM who do not respond well to traditional therapies.

## Additional file

Additional file 1: Effect of soluble BCMA on anti-BCMA CAR-T cells engagement with BCMA^+^ tumor cells. (DOCX 825 kb)

## Abbreviations

Auto-HSCT: Autologous hematopoietic stem cell transplantation
BCMA: B cell maturation antigen
CAR: Chimeric antigen receptor
CNS: Central nervous system
CR: Complete remission
CRS: Cytokine release syndrome
DXM: Dexamethasone
Hb: Hemoglobin
HGG: Human gamma globulin
LEN: Lenalidomide
MM: Multiple myeloma
NEC: Neutrophil
PDN: Prednisolone
PLT: Platelet
RRMM: Relapsed/refractory multiple myeloma
VEGF: Vascular endothelial growth factor
WBC: White blood cell

## References

[1] Palumbo A (2011). Multiple myeloma. N Engl J Med.

[2] Sonneveld P (2017). Management of multiple myeloma in the relapsed/refractory patient. Hematology Am Soc Hematol Educ Program.

[3] Laubach J, Garderet L, Mahindra A, Gahrton G, Caers J, Sezer O (2016). Management of relapsed multiple myeloma: recommendations of the International Myeloma Working Group. Leukemia.

[4] Kumar SK, Lee JH, Lahuerta JJ, Morgan G, Richardson PG, Crowley J (2012). Risk of progression and survival in multiple myeloma relapsing after therapy with IMiDs and bortezomib: a multicenter international myeloma working group study. Leukemia.

[5] Kochenderfer JN, Somerville RPT, Lu T, Yang JC, Sherry RM, Feldman SA (2017). Long-duration complete remissions of diffuse large B cell lymphoma after anti-CD19 chimeric antigen receptor T cell therapy. Mol Ther.

[6] Lee DW, Kochenderfer JN, Stetler-Stevenson M, Cui YK, Delbrook C, Feldman SA (2015). T cells expressing CD19 chimeric antigen receptors for acute lymphoblastic leukaemia in children and young adults: a phase 1 dose-escalation trial. Lancet.

[7] Maude SL, Frey N, Shaw PA, Aplenc R, Barrett DM, Bunin NJ (2014). Chimeric antigen receptor T cells for sustained remissions in leukemia. N Engl J Med.

[8] Turtle CJ, Hanafi LA, Berger C, Gooley TA, Cherian S, Huecek M (2016). CD19 CAR-T cells of defined CD4+:CD8+ composition in adult B cell ALL patients. J Clin Invest.

[9] Ali SA, Shi V, Maric I, Wang M, Stroncek DF, Rose JJ (2016). T cells expressing an anti-B-cell maturation antigen chimeric antigen receptor cause remissions of multiple myeloma. Blood.

[10] Brudno JN, Maric I, Hartman SD, Rose JJ, Wang M, Lam N (2018). T cells genetically modified to express an anti-B-cell maturation antigen chimeric antigen receptor cause remissions of poor-prognosis relapsed multiple myeloma. J Clin Oncol.

[11] Carpenter RO, Evbuomwan MO, Pittaluga S, Rose JJ, Raffeld M, Yang S (2013). B-cell maturation antigen is a promising target for adoptive T-cell therapy of multiple myeloma. Clin Cancer Res.

[12] Warsame R, Yanamandra U (2017). POEMS syndrome: an enigma. Curr Hematol Malig Rep.

[13] Nozza Andrea (2017). POEMS SYNDROME: AN UPDATE. Mediterranean Journal of Hematology and Infectious Diseases.

[14] Dispenzieri A, Kyle RA, Lacy MQ, Rajkumar SV, Therneau TM, Larson DR (2003). POEMS syndrome: definitions and long-term outcome. Blood.

[15] Dispenzieri A (2007). POEMS syndrome. Blood Rev.

[16] Lee DW, Gardner R, Porter DL, Louis CU, Ahmed N, Jensen M (2014). Current concepts in the diagnosis and management of cytokine release syndrome. Blood.

[17] Liu D, Zhao J (2018). Cytokine release syndrome: grading, modeling, and new therapy. J Hematol Oncol.

[18] Durie BG, Harousseau JL, Miguel JS, Bladé J, Barlogie B, Anderson K (2006). International uniform response criteria for multiple myeloma. Leukemia.

[19] Melchor L, Brioli A, Wardell CP, Murison A, Potter NE, Kaiser MF (2014). Single-cell genetic analysis reveals the composition of initiating clones and phylogenetic patterns of branching and parallel evolution in myeloma. Leukemia.

[20] Bolli N, Avet-Loiseau H, Wedge DC, Van Loo P, Alexandrov LB, Martincorena I (2014). Heterogeneity of genomic evolution and mutational profiles in multiple myeloma. Nat Commun.

[21] Paíno T, Paiva B, Sayagués JM, Mota I, Carvalheiro T, Corchete LA (2015). Phenotypic identification of subclones in multiple myeloma with different chemoresistant, cytogenetic and clonogenic potential. Leukemia.

[22] Ruella M, Barrett DM, Kenderian SS, Shestova O, Hofmann TJ, Perzaaelli J (2016). Dual CD19 and CD123 targeting prevents antigen-loss relapses after CD19-directed immunotherapies. J Clin Invest.

[23] Zah E, Lin MY, Silva-Benedict A, Jensen MC, Chen YY (2016). T cells expressing CD19/CD20 bispecific chimeric antigen receptors prevent antigen escape by malignant B cells. Cancer Immunol Res.

[24] Hegde M, Mukherjee M, Grada Z (2016). Tandem CAR T cells targeting HER2 and IL13Rα2 mitigate tumor antigen escape. J Clin Invest.

[25] Sanchez E, Li M, Kitto A, Li J, Wang CS, Kirk DT (2012). Serum B-cell maturation antigen is elevated in multiple myeloma and correlates with disease status and survival. Br J Haematol.

[26] Ghermezi M, Li M, Vardanyan S, Harutyunyan NM, Gottlieb J, Berenson A (2017). Serum B-cell maturation antigen: a novel biomarker to predict outcomes for multiple myeloma patients. Haematologica.

[27] Cohen AD, Garfall AL, Stadtmauer EA, Lacey SF, Lancaster E, Vogl DT (2017). Safety and efficacy of B-cell maturation antigen (BCMA)-specific chimeric antigen receptor T cells (CART-BCMA) with cyclophosphamide conditioning for refractory multiple myeloma (MM). Blood.

[28] Berdeja JG, Lin Y, Raje N, Munshi N, Siegel D, Liedtke M (2017). Durable clinical responses in heavily pretreated patients with relapsed/refractory multiple myeloma: updated results from a multicenter study of bb2121 an -BCMA CAR T cell therapy. Blood.

[29] Fan F, Zhao W, Liu J, He A, Chen Y, Cao X (2017). Durable remissions with BCMA-specific chimeric antigen receptor (CAR)-modified T cells in patients with refractory/relapsed multiple myeloma. J Clin Oncol.

[30] Hermanson DL, Barne BE, Rengarajan S, Codde R, Wang X, Tan Y (2016). A novel BCMA-specific, centyrin-based CAR-T product for the treatment of multiple myeloma. Blood.

