# Supplementary material for: Anti-BCMA CAR-T cells for treatment of plasma cell dyscrasia: case report on POEMS syndrome and multiple myeloma
Source: J Hematol Oncol. 2018 Oct 22;11:128. doi: 10.1186/s13045-018-0672-7 (PMC6198365; doi:10.1186/s13045-018-0672-7)
Supplement: Supplementary file 1 — Effect of soluble BCMA on anti-BCMA CAR-T cells engagement with BCMA+ tumor cells. (DOCX 825 kb) [file 13045_2018_672_MOESM1_ESM.docx]

**Supplemental Methods**

**Cytotoxicity Assay**

RPMI8226 and U266 (target cells, BCMA+ multiple myeloma cell lines) were co-cultured with anti-BCMA CAR-T cells (effector T cells, 25:1) in duplicate wells of a 96 well round bottom plate in AIM-V medium + 5% human serum. Graded concentrations (0, 2.5, 25ng/ml) of BCMA were added to assess the effect of soluble BCMA on CAR function. The plates were incubated at 37°C for 4 or 20 hours. Following the incubation, the cytotoxicity was determined at 4 hours by Calcein AM staining. ELISAs for IFN γ were performed using standard methods (Pierce) at 20 hours, and the results are displayed as the mean +/− the standard error of the mean.

**Supplemental Figure**

**Figure 1**

**
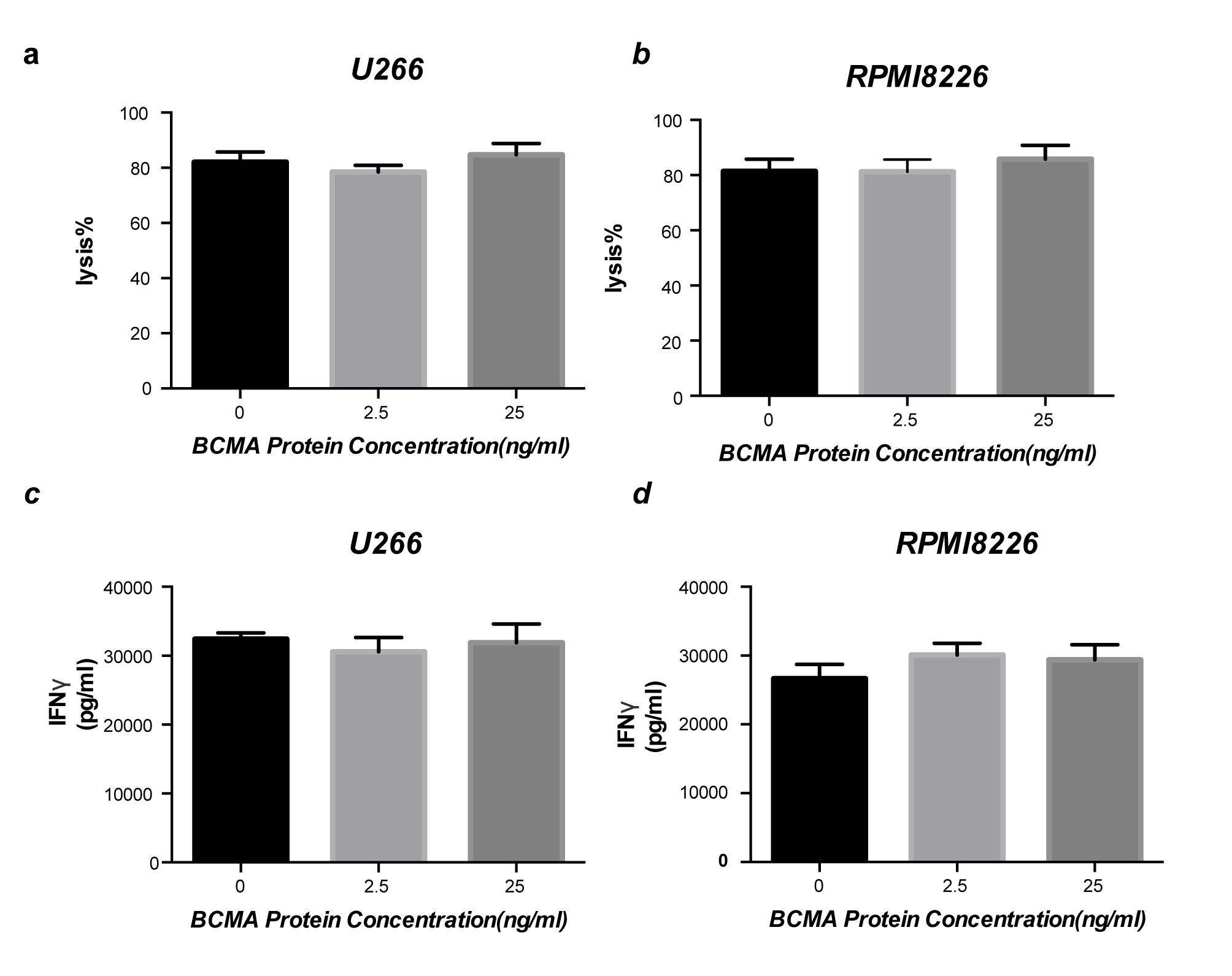
**

**Figure 1. BCMA protein in solution does not block recognition of BCMA+ target cells by Anti-BCMA CAR-T cells in vitro****.**  Anti-BCMA CAR-T cells were co-cultured with U266 and RPMI8226 cells in medium containing the indicated concentrations of BCMA protein. (a/b) The tumoricidal efficiency of anti-BCMA CAR-T cells in 4-hour cytotoxicity assay. (c/d) The level of IFN γ in culture medium after 20 hours
